# Obstructive sleep apnea: What is an orthodontist’s role?

**DOI:** 10.1186/s40510-024-00524-4

**Published:** 2024-07-01

**Authors:** Robert H. Kazmierski

**Affiliations:** Solo Private Orthodontic Practice, 110 Marter Ave., Suite 404, Moorestown, NJ 08057 USA

**Keywords:** Obstructive sleep apnea, Orthognathic surgery, Maxillomandibular advancement, Mandibular advancement devices, Palatal expansion

## Abstract

**Background:**

The American Association of Orthodontists white paper on obstructive sleep apnea and orthodontics remains the most authoritative statement on the topic. This was produced in 2019 due to increasing orthodontic interest in obstructive sleep apnea (OSA) and the lack of formal guidelines for orthodontists. Since the white paper’s release, advocacy for contrarian ideas and practices remain. Orthodontists are sometimes acting as primary care providers for OSA. Procedures appropriate only for screening are sometimes being used for diagnosis. The side effects of effective treatments such as mandibular advancement devices need further consideration. Also, research has clarified the effectiveness and ineffectiveness of treatments such as palatal expansion.

**Results:**

Part of an orthodontist’s role is screening for OSA. The correct action when this is suspected remains referral to the appropriate physician specialist for diagnosis and treatment or coordination of treatment. Orthodontists may participate in the treatment of patients with OSA as a member of a multi-disciplinary team. Effective orthodontic treatments may include orthognathic surgery with maxillomandibular advancement and mandibular advancement devices. The negative effects of the latter make this a choice of last resort. Current research indicates that OSA alone is not sufficient indication for palatal expansion.

**Conclusions:**

Orthodontists should appropriately screen for obstructive sleep apnea. This may be done as part of our health histories, our clinical examination, and review of radiographs taken for purposes other than the diagnosis and screening for OSA. Orthodontic treatment for OSA can be helpful and effective. However, this may be done only after referral to the appropriate physician specialist, as part of a multi-disciplinary team, with consideration of the likely effectiveness of treatment, and after all likely and potential negative consequences have been considered and thoroughly discussed with the patient.

## Introduction

The American Association of Orthodontists (AAO) white paper on obstructive sleep apnea (OSA) and orthodontics stands as the definitive statement on this subject [1]. Released in 2019, it was prompted by the growing interest among orthodontists in OSA and the absence of established guidelines specific to orthodontic practice [1]. However, since its publication, there persists a prevalence of advocacy for divergent ideas and practices. Some orthodontists have assumed roles akin to primary care providers for OSA, occasionally employing procedures meant for screening purposes for diagnosis. Furthermore, while effective treatments like mandibular advancement devices MADs have shown promise, their potential side effects warrant further exploration [2]. Additionally, recent research has shed light on both the potential efficacy and the limitations of treatments such as palatal expansion for OSA [3–5].

Consequently, this commentary seeks to delve into the appropriate delineation of an orthodontist's role in OSA treatment. This role can be anchored in three fundamental principles:

### First principle: do no harm

Obstructive sleep apnea (OSA) is a prevalent disorder associated with an increased risk of serious cardiovascular conditions such as coronary artery disease, heart attack, and heart failure [6]. If left inadequately managed, OSA can even lead to premature death [6]. As orthodontists, venturing into life-or-death scenarios is unfamiliar terrain for us. Therefore, when OSA is suspected or diagnosed, careful considerations must be made.

The primary healthcare provider most qualified to diagnose and guide the management of OSA patients is the physician specializing in sleep medicine, commonly known as a sleep specialist [7]. The diagnosis and management of OSA fall distinctly outside the scope of orthodontics. Without the guidance of a sleep specialist, orthodontists risk violating the principle of "do no harm."

This does not imply that orthodontists are precluded from screening for OSA. Orthodontists can indeed conduct screening assessments for OSA; however, their role is strictly delineated by the directives and recommendations provided by sleep specialists [1]. Also, orthodontists bear the responsibility of ensuring that any management provided under the guidance of a sleep specialist is efficacious, that patients are fully informed about potential side effects, and that alternative treatment options have been thoroughly explored.

Polysomnography (PSG) stands as the gold standard for diagnosing obstructive sleep apnea (OSA), providing a direct measure of apneic and hypopnea events over a specified period, known as the apnea–hypopnea index (AHI) [8]. Its validity has been endorsed by the American Academy of Pediatrics, particularly for children and adolescents exhibiting snoring or OSA symptoms [9]. PSG offers a noninvasive means of assessment [10].

In contrast, orthodontic records are not considered definitive diagnostic tools for OSA. They are viewed as indirect and less precise, primarily suitable for initial screening rather than a definitive diagnosis. For instance, cone beam computed tomography (CBCT) images, although sometimes used to assess airway dimensions, lack reliability and consistency in airway analysis [11]. Despite their potential utility in certain clinical scenarios, limitations such as static imagery, inability to evaluate airway function, and radiation risks undermine their diagnostic value [12, 13].

Similarly, lateral cephalograms, providing 2D representations of 3D structures, are less effective than CBCT in OSA screening and are unsuitable for definitive diagnosis [14]. Overall, the primary screening tools for OSA in orthodontic settings include radiographs, medical history assessment, and standardized questionnaires like the STOP-BANG for adults and the Pediatric Sleep questionnaire for children [15]. Additionally, the Modified Mallampati assessment for the palatine tonsils contributes to the screening [16].

It's important to acknowledge the evolving relevance of these screening tools in orthodontic research and their role in advancing OSA research. However, their limitations and the need for rigorous validation underscore the importance of exercising caution and considering complementary diagnostic modalities when assessing patients for OSA. Unfortunately, much of the relevant orthodontic research assessing the efficacy of appliance systems in improving airway and breathing fails to incorporate definitive diagnosis of airway issues using polysomnography (PSG) before treatment or symptom assessment post-treatment. This raises significant concerns regarding the effectiveness of these appliances in curing obstructive sleep apnea (OSA) or any airway-related issues.

For example, it may seem intuitive that an expander could increase airway dimensions as observed in CBCT scans or lateral cephalograms [17]. However, it cannot be definitively concluded that this treatment modality contributes to OSA treatment success utilizing measurements of dimensional changes alone. The crucial question arises: what impact does the change in airway volume have on OSA treatment if the patient did not initially have OSA, or if research fails to establish a definitive difference in pre- and post-treatment apnea–hypopnea index (AHI)?

The use of PSG appears necessary to validate treatment with orthodontic appliances for airway improvement in OSA patients. The additional requirement of having a control group seems critical in pediatric patients, as their developing hard and soft tissues undergo constant change. With pediatric patients, observing the effects of an orthodontic treatment protocol may frequently provide no value without a comparison to a control group. A control group would elucidate whether claimed airway improvements are attributable to treatment rather than natural growth. A third time point sufficiently past treatment completion seems necessary with pediatric patients as well. Even if a post treatment difference is achieved, growth may result in the control group catching up. This would nullify any initial difference from treatment.

In summary, for more robust and valid airway clinical and research practices, PSG assessment before and after treatment is essential to ascertain treatment efficacy.

### Second principle: soft tissue changes do not necessarily follow hard tissue changes

The principle that soft tissue changes do not necessarily follow hard tissue changes is significant, particularly in the context of viewing obstructive sleep apnea (OSA) primarily as a soft tissue or neuromuscular issue rather than a hard tissue problem [18]. The presence of hypoxemia serves as a critical factor in distinguishing between these etiologies, with constant hypoxemia indicating a narrowing in the hard tissue boundaries of the airway [19].

Given that most orthodontic treatments focus on adjusting hard tissues, there's often an expectation that neuromuscular tissues will adapt accordingly. However, it's important to acknowledge that the adaptation of soft tissues to these changes can sometimes be unpredictable. Hence, it's crucial to understand which hard tissue changes might have a predictable effect on the relevant neuromuscular tissues.

For example, surgical maxillomandibular advancement (MMA) appears to yield the most reliable and predictable outcomes in terms of soft tissue adaptation for OSA patients [20]. A recent meta-analysis assessing the apnea–hypopnea index (AHI) post-MMA surgery reported promising results, with 85% of patients considered surgical successes and 38.5% completely cured of OSA [21]. These findings underscore the potential of maxillomandibular advancement surgery as a viable cure for OSA.

In contrast, mandibular advancement devices (MAD) aim to advance the mandible non-surgically by stretching ligaments and musculature around the jaws. While MADs may offer benefits in terms of airway improvement, their effectiveness may not be as reliable or predictable as surgical interventions like MMA [22]. Therefore, mandibular advancement devices (MADs) might offer improvement in airway problems by constantly increasing airway diameter and maintaining soft tissue displacement. However, it's important to note that the adaptation of soft tissues to mandibular jaw displacement is not guaranteed. Also, long-term use of MADs has been associated with various malocclusions and their efficacy in reducing the apnea–hypopnea index (AHI) in adults is reported to be moderate over the long term [22]. Hence, caution must be exercised when prescribing these appliances as a treatment for OSA, and patients should be informed of the potential risks involved.

Regarding palatal expansion, it is logical to assume that it might increase the nasopharyngeal airway space and consequently decrease nasal airway resistance. However, the available evidence supporting this is only short-term, equivocal, and of low quality [23, 24]. While a recent meta-analysis has shown a statistically significant reduction in nasal resistance following palatal expansion [25], it can be argued that increasing the palatal width and nasopharyngeal airway space may not necessarily affect the collapsibility of the pharyngeal airway or the neuromuscular tone during sleep, both of which could be causative factors in a patient’s OSA [26]. It's important to note that the nasopharynx is not directly connected to the non-collapsible trachea; rather, the collapsible pharyngeal airway lies between these two structures. Therefore, palatal expansion and the resulting increase in nasopharyngeal space may not directly impact the collapsibility of the pharyngeal airway. Research on the effects of expansion on the collapsibility of the pharyngeal airway space is lacking. Moreover, improvement in the patency of the nasal airway does not necessarily translate to an improvement in the patient's AHI. Consequently, few studies have investigated the long-term changes in AHI post-expansion. Any post-palatal expansion long-term change in AHI should be viewed as a fortunate outcome rather than a predictable one, as it is often short-term and non-predictable.

For pediatric patients, the situation is considerably less ambiguous. Research conducted on pediatric patients with OSA has indicated that watchful waiting has an equivalent effect on any change in the apnea–hypopnea index (AHI) as palatal expansion [5]. It appears that studies utilizing polysomnography to assess AHI in pediatric patients do not endorse the use of palatal expansion for the treatment or prevention of OSA [27]. Instead, the use of palatal expanders is advocated solely for orthodontic clinical purposes [27].

Within this second principle and preceding this point, orthodontic treatments for OSA vary in their success rates, ranging from highly successful to occasionally successful within specific parameters and circumstances. Regrettably, some treatments that are known to be ineffective need to be addressed. Among these is the misconception regarding the influence of orthodontic extractions on the airway. To put it plainly, there is no scientific evidence available to support the notion that a cause-and-effect relationship exists between orthodontic extractions and OSA [28]. Since orthodontic extractions do not cause OSA, it logically follows that opening prior extraction spaces is not a legitimate treatment for OSA either. Similarly, there is no scientific evidence supporting the effectiveness of treatments to increase "tongue space" for the treatment of OSA.

Overall, we must acknowledge that logical deductions can become invalid when contradicted by valid research evidence. Many concepts may initially seem plausible but are ultimately proven false when subjected to rigorous research scrutiny. An analogy can be drawn between the avoidance of orthodontic extractions to prevent OSA, treatments to increase "tongue space" for the treatment of OSA, and the historical medical practice of bloodletting to treat various ailments. Due to misplaced trust in personal clinical experiences, numerous success stories, and detailed theoretical explanations, bloodletting remained a medically accepted practice for over 2,000 years. It was only through research demonstrating its lack of benefit that the practice was ultimately discontinued. Similarly, in orthodontics, we must prioritize evidence-based approaches over anecdotal experiences or theoretical explanations. Valid research should serve as the guiding principle in determining the most effective treatments for OSA and other conditions.

### Third principle: selection of treatment with least undesirable side effects, acceptable to patients

Indeed, the selection of treatment for obstructive sleep apnea (OSA) involves a delicate balance considering various factors unique to each patient and situation. While positive pressure appliances (PPA) and weight loss are commonly recommended for adult patients [29], adenotonsillectomy is often favored for pediatric patients over two years old [30]. However, when dental interventions come into play, maxillomandibular advancement (MMA) emerges as a successful option [21], albeit with limited acceptability due to its surgical nature. Mandibular advancement devices (MAD) offer a non-surgical alternative, but their long-term efficacy relies on consistent nightly wear. This and their dental side effects can make them less desirable for some patients [31]. It's crucial to acknowledge that while MADs can provide ongoing symptom relief, they are not a definitive cure for OSA.

There would seem little risk of side effects when MADs are used temporarily as a substitute for CPAP, particularly during travel, or as part of a surgery-first treatment plan while awaiting MMA. However, when they are worn indefinitely as a definitive treatment, mandibular advancement devices (MAD) exert significant ongoing forces on the teeth, potentially leading to unintended side effects and worsening of the occlusion [31]. While these appliances may have some corrective effect in cases of class II malocclusion, their prolonged use can result in detrimental effects on the periodontium and dental occlusion [31]. It's crucial to recognize that while MADs may offer temporary relief for obstructive sleep apnea (OSA), their continued use as a long-term solution poses risks to the health of the joint and dentition. Prolonged use of MADs can lead to anterior flaring of the mandibular incisors, resulting in class III malocclusions and other occlusal abnormalities [31]. Additionally, MADs can contribute to conditions such as anterior edge-to-edge relationships, posterior open bites, and anterior open bites, which can further compromise dental health and stability [31].

In my own clinical experience, I have observed cases where MAD appliances have caused significant dental changes, including flaring of mandibular incisors and associated lingual alveolar bone atrophy, ultimately leading to tooth loss. Once these changes occur, orthodontic correction may be challenging or even impossible, highlighting the irreversible nature of the damage caused by MADs.

Titration of the mandibular advancement in mandibular advancement devices (MADs) has been used to minimize the impact of orthodontic forces [32]. Morning occlusal guides are sometimes used to reverse the effects of MAD wear as well [33]. However, long-term damage may still occur, highlighting the importance of careful monitoring and consideration of alternative treatments.

As orthodontists, it's essential to exercise caution when considering MADs as a treatment for OSA, emphasizing the potential risks and limitations to patients. Long-term use of MADs should be avoided, and alternative treatment options should be explored whenever possible to minimize the risk of adverse dental outcomes.

In cases where negative side effects of MADs become evident, transitioning to continuous positive airway pressure (CPAP) therapy or considering maxillomandibular advancement (MMA) surgery may be necessary to address the issues effectively. Given the eventual dental effects, it is concerning that some patients may choose MADs without being adequately informed of the potential risks and limitations. During my clinical experience, I have sadly encountered patients seeking orthodontic solutions for the negative outcomes of MADs who were unaware of these possibilities and had not received comprehensive guidance from a physician specializing in sleep medicine or their orthodontists. This underscores the importance of thorough patient education and shared decision-making when considering treatment options for OSA. Patients should be fully informed of the potential benefits and risks associated with each treatment modality, allowing them to make informed decisions about their care in collaboration with healthcare providers specializing in sleep medicine.

It is important to recognize that while mandibular advancement devices (MADs) may pose risks and potential harms, there are situations where they can be a viable and appropriate treatment option for patients with obstructive sleep apnea (OSA). Despite their drawbacks, MADs may be the best available choice for certain individuals who are not suitable candidates for other treatments or who are unable to tolerate alternative interventions. For instance, in cases where a patient's health is at risk due to untreated OSA and they cannot undergo or tolerate other treatment modalities, using a MAD may be justified. In such circumstances, the potential benefits of MAD therapy may outweigh the risks and limitations. Ultimately, the decision to use a MAD should be made on a case-by-case basis, considering the individual patient's medical history, preferences, and treatment goals. It is crucial for healthcare providers to thoroughly discuss the potential benefits, risks, and alternatives with patients, empowering them to make informed decisions about their care in collaboration with their healthcare team.

Every treatment option carries its own set of benefits and limitations, and the optimal choice depends on the patient's preferences, medical history, and willingness to undergo certain procedures. As orthodontists, we must carefully weigh these factors to provide personalized treatment plans that prioritize both effectiveness and patient acceptance. Upon evaluating the spectrum of treatments for obstructive sleep apnea (OSA), a distinct trend emerges. When arranged in descending order of preference, orthodontic interventions consistently rank lower on the list. While maxillomandibular advancement (MMA) surgery proves effective, its association with orthognathic surgery often deters patients from opting for this approach. In contrast, mandibular advancement devices (MADs) demonstrate efficacy for many patients. However, their eventual adverse effects relegate them to a last-resort status. The remaining orthodontic treatments for OSA either lack sufficient reliability or are contradicted by robust research findings.

This pattern underscores the importance of a discerning approach to treatment selection. By prioritizing interventions with the optimal balance of effectiveness and tolerability, healthcare providers can guide patients toward solutions that offer the greatest benefit with the fewest drawbacks.

## Conclusion

In summary, the role of an orthodontist in managing obstructive sleep apnea (OSA) should involve thorough screening procedures that incorporate medical history, physical examination, relevant sleep apnea questionnaires, and clinical assessments supported by radiographic findings. Subsequent referral to a sleep medicine specialist, preferably a physician board-certified in sleep medicine (PBCSM), is essential to confirm the diagnosis.

Once the diagnosis is confirmed, treatment decisions should be made in collaboration with the PBCSM, prioritizing the most effective and least harmful evidence-supported options available. This approach ensures that patients receive comprehensive care that addresses their OSA condition while minimizing risks and optimizing outcomes.

## Data Availability

Not Applicable.

## Abbreviations

AHI: Apnea–hypopnea index
CBCT: Cone beam computed tomography
CPAP: Continuous positive airway pressure
CPR: Cardiopulmonary resuscitation
MAD: Mandibular advancement devices
MMA: Maxillomandibular advancement
OSA: Obstructive sleep apnea
PBCSM: Physician board-certified in sleep medicine
PPA: Positive pressure appliances
PSG: Polysomnography
SARME: Surgically assisted rapid maxillary expansion
